# Case Report: Stem cell therapy in amyotrophic lateral sclerosis

**DOI:** 10.12688/f1000research.73967.1

**Published:** 2021-10-25

**Authors:** Ala'a A. Hassan, Jeananne Elkins, Hisham Y. Hassan

**Affiliations:** 1 Physiotherapy and Rehabilitation Department, Bahrain Defence Force Hospital, Riffa, Bahrain; 2 Northeastern University, Boston, Massachusetts, USA; 3 Banoon ART and Cytogenetics Center, Bahrain Defence Force Hospital, Riffa, Bahrain

**Keywords:** Amyotrophic lateral sclerosis, stem cell therapy, physical therapy, rehabilitation

## Abstract

Amyotrophic lateral sclerosis (ALS) is a progressive motor neuron disease leading to loss of upper and lower motor neurons at both spinal and bulbar levels.^^ For patients with ALS rehabilitation is important to maintain functional independence, ensure safety and optimize quality of life but is not curative. Stem cell therapy (SCT) provides a new approach to treat previously incurable diseases although peer reviewed published evidence has shown no benefit in ALS for slowing disease progression or functional loss.

This case report presents a patient with ALS who underwent SCT but deteriorated rapidly after the procedure. Whether the deterioration was due to the natural progress of the disease or expedited by SCT remains unknown. The ethical considerations of how marketing influences healthcare and individuals’ decisions in desperate situations along with reasons for taking desperate measures are discussed.  Patient education and open communication with ALS patients are imperative in gaining patient satisfaction and overcoming ill effects that marketing could have on unconventional methods of intervention. Raising awareness about the availability and access to multidisciplinary care, the timing of decisions with regards to symptom management and end of life care have proven to enhance the quality of life for such patients.

## Introduction

Amyotrophic lateral sclerosis (ALS), an incurable and fatal motor neuron disorder, leads to progressive destruction and loss of upper and lower motor neurons at both spinal and bulbar levels. ^1^ Worldwide, two to five cases per 100,000 individuals per year are diagnosed with ALS. ^2^^-^^4^ The average age of onset of ALS is between 50-65 years old, and it is more common in men. ^1^ The cause of the familial type of ALS is mutations in several genes. ^1^ The cause of sporadic cases which represent 90-95% of all cases is largely unknown, but most probably includes a combination of environmental and genetic factors. ^2^ The symptoms occur in a gradual and sporadic pattern with progressive deterioration resulting in an average lifespan of 3-5 years after diagnosis. ^5^ By the time the disease becomes noticeable, almost 80% of motor neurons have been lost. ^3^ The most common symptoms are distal or proximal muscle weakness of upper and lower limbs, fasciculation and spasticity. ^1^^,^^3^ Once bulbar levels are affected then dysphagia and dysarthria become apparent leading to weight loss and anorexia. ^1^^,^^3^ At advanced stages of the disease, the respiratory system is affected leading to respiratory failure with the need for assisted ventilation to sustain life. ^1^^,^^3^

The only Federal Drug Administration (United States) approved drug for ALS, Riluzole, reduces the disease progression and improves the survival by only 3-6 months. ^3^^,^^5^ Therefore, ALS is managed predominantly through supportive care and comprehensive multidisciplinary management. ^5^^,^^6^ Considering the progressive nature of the disease, patients are tempted to seek any available intervention to modify or cease the progression of the disease. Despite the controversy associated with its efficacy and ongoing research, stem cell therapy is one of the few available interventions attempted for ALS. ^7^

## Case presentation

A 52-year-old Arab male working as a military officer and married with three children presented to the neurology outpatient clinic in October 2016. His past medical history revealed appendectomy (2012) and colon cancer (2013). Family history was clear and not known for any genetic diseases. His initial symptoms were weakening of the right upper limb associated and painful spasms. He did not have other symptoms in the neural axis and was functionally independent. He scored 126 points on the Functional Independence Measure (FIM) ^8^ and 24 points on the Dynamic Gait Index (DGI) ^9^ indicating he was functionally independent and had excellent balance with no risk of falls. However, electrophysiological testing confirmed ALS. At the early stage of ALS his right upper limb showed significant atrophy in comparison to the left side with clear spontaneous fasciculation involving the deltoid and pectoralis muscles. His reflexes were exaggerated with positive Hoffman signs but examination of other limbs was unremarkable. The patient was referred to physical therapy in October 2016 to maintain his functional level and activity. Physical rehabilitation focused on general strengthening exercises at moderate intensity, balance and plyometric training and aerobic training. He received therapy 3 times a week for 8 weeks before experiencing further functional decline.

In the following 6-8 months, the ALS progressed and the patient’s function declined. He lost his ability to grip objects and developed a shuffling gait pattern but was still independent. He was independent in transfers; however, he was falling more often. The FIM score declined to 96 points indicating he required minimal to moderate assistance with activities of daily living. ^8^ His DGI declined to 10 points categorizing him at high risk of falls. ^9^

At this point in time, the patient began considering undergoing stem cell therapy (SCT) because of his declining function. Educational information about how SCT is still in the research phase and that no conclusive evidence for effectiveness in ALS has been established was provided to the patient. However, the patient had confirmed his decision to undergo SCT in Ukraine. Per the patient reports, he received SCT on two consecutive days. The first day consisted of an intravenous drip-fed administration of 2.1 ml of embryonic stem cells. The second day subcutaneous implantation of the 5.1 ml of embryonic stem cells in the frontal abdominal wall was administered. Of note, the SCT were negative from all bacterial, viral and intracellular infections.

Comparing this sample to the established human clinical trials published in Goutman *et al*, ^10^ no trial used embryonic stem cells and the intraspinal route of administration was more common than the intravenous route. No mention of immunosuppression medications was indicated in the patient report. Although the consensus regarding the dosage of stem cells is variable and inconclusive, the amount of stem cells administered in the studies ranged between 300-600 micrograms/day over 5-6 days; thus, this patient’s dosage does not match that found in the literature. ^11^ Moreover, the patient did not receive a follow-up session after undergoing the procedure.

Six months post SCT, he had lost functional ability and was primarily wheelchair bound. He was still able to walk with a gutter frame with assistance for a distance of 10-20 meters before becoming fatigued. He was dependent on his family for his basic activities of daily living and was at high risk of falls (FIM Score 86 points ^8^ and DGI 4 points ^9^). Physical therapy focused on maintaining the available function and strength at this stage through functional training of lower limbs including sit-to-stand exercises, practicing transfers and gait training. Upper limb and balance training were eliminated as the patient lost complete muscle power and ability. The treatment changed session by session based on the patient’s physical ability and psychological wellbeing. The patient died in March 2021 at the age of 57 years - 5 years from the date of the diagnosis in 2016.

## Discussion

The question of whether SCT expedited this deterioration or whether this functional decline is part of the natural disease course remains unanswered. There is a lack of confirmatory evidence that exercise training during physical therapy sessions causes deterioration in patient condition. Exercises focusing on strength, endurance and aerobic training at moderate intensity are not associated with delaying or expediting the disease process. However, over-exertion should be avoided as it can induce muscle damage in addition to fatigue and pain. ^5^^,^^12^

The main characteristics of stem cells are self-renewal, proliferation and full differentiation into the needed cell type. ^13^ Therefore, SCT provides a new horizon to attempt to treat the incurable diseases although no confirmative evidence has been established yet. The aims of SCT in neurological conditions are to replace the lost or damaged nerve cells, restore the homeostasis of the central nervous system, protect the unaffected surrounding tissues, and enhance the endogenous repair processes initiated by the body. ^13^ Trials of SCT for ALS, Parkinson’s disease, stroke, spinal cord injury and epilepsy have been primarily carried out in animals, ^11^ but extrapolating results to human trials is still challenging.

The major challenge with SCT in ALS is that the pathophysiological mechanisms of ALS are varied including mitochondrial impairment, oxidative stress, neuronal cytotoxicity, altered gene expression and cell death. ^3^ Consequently, to decipher the exact mechanisms responsible for disease onset and progression is mysterious, and to implement a treatment plan aiming to target these multiple mechanisms becomes even more challenging. ^10^^,^^13^ Goutman *et al* ^10^ contend with SCT there are no clear pharmacokinetic and pharmacodynamics markers thus making it difficult to decipher its effectiveness or adverse reactions. This also poses the challenge of ensuring the graft’s survival, and the outcome measures used in ALS patients, such as ALS Functional Rating Scale-Revised, ^14^ reflect on the functional status of a patient, but not on the motor neuron environment. Hence, the efficacy of SCT and new motor neuron formation remains unknown. The outcome measures used in ALS research must be sensitive to capture changes in the course of the disease. ^4^

ALS disease, like many other neurological diseases, has variable rates of progression among patients. The type of ALS, bulbar or spinal, has an effect on the prognosis. Questions regarding the dosage of SCT and type of the most effective stem cell in this patient population remain unknown and require further research. Would it be beneficial to administer SCT in the early or late stages of ALS? Is there a criterion for patient selection for ALS? More research is required to identify the effective protocol for SCT in this patient population.

This patient is truly an example of how desperate people take desperate measures and make desperate choices even though those measures could potentially worsen their quality of life. Being a burden on the family is a major concern for patients and a reason for these desperate decisions. Additionally, neither patients nor their families may be ready for the “final stage” i.e. facing death. Uncertainty of timing may be another reason and accepting the medical condition may be troublesome for some patients and their family members. ^10^ Moreover, undergoing surgeries or novel procedures including SCT could provide patients and their families with reassurance that they have exhausted all the interventional options regardless of the outcome. ^12^

## Ethical and clinical recommendations

Patient education and communication reduces anxiety and fear among patients. The way the diagnosis is communicated at the first step determines the doctor-patient relationship. Spending adequate time discussing the diagnosis is a predictor of higher patient satisfaction with medical care. ^12^ Discussing the diagnosis in the presence of family members and arranging regular meetings after the first session to address further concerns reduces patient anxiety. ^12^ Lack of or inappropriate communication from the physicians and healthcare workers can lead to patients searching for and locating alternative methods of intervention. ^12^ In addition, many patients are unaware of evidence-based medicine and may be attracted to marketing campaigns.

Specifically for patients with ALS there are three critical points in healthcare. These are the timing and delivery of the diagnosis, the availability and access to multidisciplinary care, and the timing of decisions with regards to symptom management and end of life care. ^15^ This standard of care has resulted in improved quality of life for patients, increased survival and reduced hospital admission or length of stays. ^15^ Hogden *et al* ^15^ suggest palliative care for patients with ALS is appropriate from the time of diagnosis and the involvement of the palliative care specialist should fluctuate based on the patient and family’s needs as the disease progresses.

Technological advancements in healthcare have worked immensely to prolong terminally ill patients’ lives through methods including mechanical ventilation, tracheostomy, and feeding tubes. However, while these advancements can prolong lives, they do not enhance the quality of life in fragile patients. ^12^ Focusing on mental well-being, providing comfort, minimizing pain, enjoying quality time with family and focusing at the participation level of function are essential to improve quality of life. ^12^ Group therapy could also provide social support and promote added benefits. ^16^

The impact of marketing on healthcare decisions as well as how companies may take advantage of terminally sick patients and their families is often underestimated. Many companies extoll the benefits of SCT whether for the sake of gaining profit or having participants to give their consent for human clinical trials. Patients may believe if they agree to undergo a clinical trial, they will be receiving the best medical care available or care that is not available to them with their local physician or specialist. ^10^ Marketers advocate that patients are consumers of healthcare services and it is their absolute right to select whatever treatment they want even if the knowledge available regarding the intervention is not conclusive. ^10^ Under this approach, marketers sell their products by purporting to respect the patient’s interest and offer the patient as being the expert to weigh the risks against the benefits.

Although technological advancements and interventions such as SCT provide hope for complex neurological conditions, controversial scientific evidence disproving their efficacy could impede recommending them to patients. The strength of this paper is in highlighting the ethical considerations that are associated with clinical decision-making in such complicated patient conditions. Proficient clinical skills, open and transparent communication and patient education are imperative in such clinical cases despite the attempts made by marketers of healthcare companies to gain profit. The major limitation of this paper is it being a case report thereby limiting its extrapolation to other patient populations. Complex and progressive neurological conditions such as ALS have individual differences from one case to another, which makes each case management distinctive and tailored to the patient’s needs.

## Conclusion

Progressive neurological conditions including ALS are challenging from a clinician’s perspective in terms of medical management and often overwhelming from a patient and family perspective. Coping and accepting the functional deterioration as well as the inevitable death associated with ALS is difficult. Acknowledging the scientific advancements including SCT can provide hope; however, the lack of scientific evidence to prove its effectiveness in ALS imposes challenges in terms of finding a cure. Marketers advocate on these advancements tempting desperate patients to make decisions despite the inconclusive evidence. Communication and collaboration of multidisciplinary team management with the patient and family members in these situations is crucial to overcome the negative impact that marketing would have on their decision-making.

## Data availability

All data underlying the results are available as part of the article and no additional source data are required.

## Consent

Written informed consent for publication of the participant’s details was obtained from the participant’s spouse.
