# Supplementary material for: Case Report: Stem cell therapy in amyotrophic lateral sclerosis
Source: F1000Res. 2022 Jan 10;10:1080. Originally published 2021 Oct 25. [Version 2] doi: 10.12688/f1000research.73967.2 (PMC8762680; doi:10.12688/f1000research.73967.2)
Supplement: Peer review history

# Peer review: Reviewer response for version 2

**DOI:** 10.5256/f1000research.120064.r119151

**Published:** 2022-01-14

**Authors:** Ebrahim Rajab

**Affiliations:** 1 School of Medicine, Royal College of Surgeons in Ireland - Medical University Bahrain, Adliya, Bahrain

I have just one outstanding comment.  The author mentions that “I have added the age and gender of the patient with a brief about his symptom deterioration and decision to undergo SCT in the abstract.” Unless I am mistaken, I see no mention of it – it looks like version 1 to me.

Are enough details provided of any physical examination and diagnostic tests, treatment given and outcomes?

Yes

Is the case presented with sufficient detail to be useful for other practitioners?

Yes

Is sufficient discussion included of the importance of the findings and their relevance to future understanding of disease processes, diagnosis or treatment?

Yes

Is the background of the case’s history and progression described in sufficient detail?

Yes

Reviewer Expertise:

Learning and Memory

I confirm that I have read this submission and believe that I have an appropriate level of expertise to confirm that it is of an acceptable scientific standard.

# Peer review: Reviewer response for version 2

**DOI:** 10.5256/f1000research.120064.r119152

**Published:** 2022-01-11

**Authors:** Dr. Julie Sprakel

**Affiliations:** 1 Think Pink: Bahrain Breast Cancer Society, Manama, Bahrain; 2 University of São Paulo, Ribeirão Preto, Brazil

Changes add value to the text

Are enough details provided of any physical examination and diagnostic tests, treatment given and outcomes?

Yes

Is the case presented with sufficient detail to be useful for other practitioners?

Yes

Is sufficient discussion included of the importance of the findings and their relevance to future understanding of disease processes, diagnosis or treatment?

Yes

Is the background of the case’s history and progression described in sufficient detail?

Yes

Reviewer Expertise:

Breast Cancer, Guideline Development,

I confirm that I have read this submission and believe that I have an appropriate level of expertise to confirm that it is of an acceptable scientific standard.

# Peer review: Reviewer response for version 1

**DOI:** 10.5256/f1000research.77663.r101768

**Published:** 2022-01-04

**Authors:** Ebrahim Rajab

**Affiliations:** 1 School of Medicine, Royal College of Surgeons in Ireland - Medical University Bahrain, Adliya, Bahrain

This case report focuses on the need for amyotrophic lateral sclerosis (ALS) patients to receive evidence-based advice, education, and multidisciplinary care in a collaborative and timely fashion. The authors highlight how the marketing of stem cell therapy (SCT) by private clinics/companies can influence patient decisions even though this treatment is currently experimental and of variable success.

 Detailed points

**Abstract**

- Mention the age and gender of the patient.
- Add a sentence explaining that the patient elected to go overseas for SCT of their own accord.
- Mention that the patient received physical therapy treatment.
- Give a brief time course for deterioration of symptoms.

**Introduction section**

- The second paragraph, the final sentence should include more up-to-date references on SCT e.g. Mazzini et al. 2018 ^1^;  e.g. Morata-Tarifa et al., 2021 ^2^)
- This section should end by stating the aim/purpose of this case report more clearly.

**Case Presentation section**

- Please add a reference in the 3rd paragraph, the sentence beginning “Educational information about how SCT is still in the research phase…”.
- Did the patient and/or his family provide their own viewpoint on the SCT treatment following return from overseas? Did their viewpoint change as the ALS deteriorated?
- The authors could summarize the clinical course, FIM, and DGI results and observations in a table or figure. Also, the authors could add the stage of ALS if they feel it is appropriate.

**Discussion section**

- The first paragraph should summarise the key points of the case report and link back to the aims of the case report.
- The 2nd, 3rd and fourth paragraphs could be condensed given that their content is not the case report's primary focus. Please provide a sentence or two specifically on the benefits/issues of embryonic SCT given that this was the treatment in the present case.

**Ethical and clinical recommendations**

- Since the SCT was administered overseas, what additional ethical or clinical issues did this raise for the care team, if at all?

Are enough details provided of any physical examination and diagnostic tests, treatment given and outcomes?

Yes

Is the case presented with sufficient detail to be useful for other practitioners?

Yes

Is sufficient discussion included of the importance of the findings and their relevance to future understanding of disease processes, diagnosis or treatment?

Yes

Is the background of the case’s history and progression described in sufficient detail?

Yes

Reviewer Expertise:

Learning and Memory

I confirm that I have read this submission and believe that I have an appropriate level of expertise to confirm that it is of an acceptable scientific standard, however I have significant reservations, as outlined above.

## References

[1] : Advances in stem cell therapy for amyotrophic lateral sclerosis. *Expert Opinion on Biological Therapy* .2018;18(8) : 10.1080/14712598.2018.1503248 865-881 10.1080/14712598.2018.1503248 30025485

[2] : Looking backward to move forward: a meta-analysis of stem cell therapy in amyotrophic lateral sclerosis. *npj Regenerative Medicine* .2021;6(1) : 10.1038/s41536-021-00131-5 10.1038/s41536-021-00131-5 33795700PMC8016966

# Response

**Published:** 2022-01-06

**Authors:** Alaa Hassan

**Affiliations:** BDF Hospital, Bahrain

Thank you very much Dr. Paul for your revision and insightful recommendations and edits. I agree with most of the points you have mentioned and I have accordingly made the necessary edits. Thank you for bringing new references to my attention and I have also added these to my paper.

 However, the only two points I did not address were having the results in tables, I did not address that because the numbers for the outcome measures were done on two or three sessions, and having them within the text with a brief interpretation of what they mean would suffice. There were no other outcome measures used with this patient to have a table of results displayed.

 The other point concerning having the procedure done overseas and whether that has any ethical issues or considerations I feel that I did not have enough information about the procedure. The patient was not given enough details and reports throughout the course of the procedure and it was difficult to access information especially since he was not given any follow-up appointments. As discussed in the paper, the family supported the patient in the decision not knowing what SCT really entails and how much research is out there to support its effectiveness. They mainly supported him because this intervention was simply not available in his home country. So, not having enough information about what has happened makes it difficult for me to discuss or make any points regarding that other than focusing on the aim of this paper which was how marketing for certain interventions might actually be deceiving. 

 I thank you again for your time and valuable input which I have taken on board in making this paper worth indexing.

# Peer review: Reviewer response for version 1

**DOI:** 10.5256/f1000research.77663.r97829

**Published:** 2021-11-01

**Authors:** Dr. Julie Sprakel

**Affiliations:** 1 Think Pink: Bahrain Breast Cancer Society, Manama, Bahrain; 2 University of São Paulo, Ribeirão Preto, Brazil

It is a constructive and comprehensive piece of work. The authors take a unique look from a different standpoint when they address "ethical and clinical recommendations".

 The impact of marketing "new scientific treatments" with no confirmative evidence could be further discussed in many other research articles and begs the question of "governance".

 The authors clearly looked at a holistic picture of what can influence a patient and what lengths they are willing to go, for their health. It opens up to a further publication specifically around treatments without addressing harms and benefits.

Are enough details provided of any physical examination and diagnostic tests, treatment given and outcomes?

Yes

Is the case presented with sufficient detail to be useful for other practitioners?

Yes

Is sufficient discussion included of the importance of the findings and their relevance to future understanding of disease processes, diagnosis or treatment?

Yes

Is the background of the case’s history and progression described in sufficient detail?

Yes

Reviewer Expertise:

Breast Cancer, Guideline Development

I confirm that I have read this submission and believe that I have an appropriate level of expertise to confirm that it is of an acceptable scientific standard.

# Response

**Published:** 2021-11-02

**Authors:** Alaa Hassan

**Affiliations:** BDF Hospital, Bahrain

Thank you for your time to read and review this article.

 Thank you for your comments. I am glad that the take home message of how marketing could affect a patient's decision in pursuing treatments that lack scientific evidence is clearly portrayed to you and you agree with.
